# Exploring the impact of a chemical disinfectant and an antiviral drug for RNA virus management in the Mediterranean fruit fly mass‐rearing

**DOI:** 10.1111/1744-7917.13477

**Published:** 2024-11-29

**Authors:** Luis Hernández‐Pelegrín, Pablo García‐Castillo, Marta Catalá‐Oltra, Óscar Dembilio, Vera I.D. Ros, Salvador Herrero

**Affiliations:** ^1^ Department of Genetics Universitat de València, Valencia Spain; ^2^ Empresa de Transformación Agraria S.A., S.M.E., M.P. (TRAGSA) Paterna Spain; ^3^ Laboratory of Virology Wageningen University and Research Wageningen The Netherlands

**Keywords:** covert RNA virus, insect specific virus, mass‐rearing, sterile insect technique, virus mitigation

## Abstract

The Mediterranean fruit fly is an agricultural pest of a wide variety of fruit crops. An effective method to counteract them in the field is through the application of the sterile insect technique, which requires the mass‐production of sterile males. The presence of pathogens, and specifically viruses, threatens the well‐being of mass‐reared insects generating an interest on the development of strategies for viral elimination or containment. Thirteen RNA viruses have been described in the medfly although so far only one of them, *Ceratitis capitata* nora virus, has been associated with detrimental effects on medfly development. In this context, medfly larvae were supplied with a chemical compound (formaldehyde) and an antiviral compound (ribavirin) via oral feeding to (1) test the potential of these compounds for viral elimination and (2) analyze their effect on medfly development. Overall, formaldehyde treatment did not reduce the viral titer for any of the tested viruses, while ribavirin effectively reduced the levels of two widespread RNA viruses but not in a dose–response manner. However, the addition of both compounds correlated with detrimental effects on medfly fitness, arguing against their use in mass‐rearing facilities.

## Introduction

*Ceratitis capitata* (Diptera: Tephritidae) (Wiedemann, 1824), also known as the Mediterranean fruit fly or medfly, is a highly destructive agricultural pest that affects a wide variety of fruit crops around the globe (Tiring & Satar, 2021). Medflies are counteracted through the application of the sterile insect technique (SIT), which relies on the production and release of millions of sterile medfly males (Klassen, 2005). Sterile males are produced in medfly mass‐rearing facilities in which the presence of pathogens together with certain environmental conditions (i.e. high densities, suboptimal temperature or humidity), and the low genetic diversity of the insects, can lead to disease outbreaks and production issues (Eilenberg *et al.*, 2018). In this line, diverse viruses act as natural pathogens of insects and have been associated with disease outbreaks in commercial insect‐rearing facilities (Maciel‐Vergara & Ros, 2017; Armién *et al.*, 2023).

In the medfly, 13 RNA viruses have been described so far (Sharpe *et al.*, 2021; Hernández‐Pelegrín *et al.*, 2022) and only one of them, *Ceratitis capitata* nora virus (CcaNV), has been associated with detrimental effects on medfly development (Llopis‐Giménez *et al.*, 2017; Hernández‐Pelegrín *et al.*, 2024). Specifically, higher CcaNV levels were associated with a reduction of pupal weight and adult longevity under starvation stress (Hernández‐Pelegrín *et al.*, 2024). Even though no obvious detrimental effects have been associated with other medfly RNA viruses, some of these viruses belong to families in which certain species are known to affect insect physiology and development. For instance, members of the family *Iflaviridae* decreased larval and pupal growth rates in the cotton bollworm *Helicoverpa armigera* (Yuan *et al.*, 2020) and caused the deformity of wings in the honeybee *Apis mellifera* (Martin & Brettell, 2019).

In this context, the insect rearing industry needs to develop efficient methods to maintain insect colonies free of potentially pathogenic viruses. The methods currently applied to prevent and mitigate pathogen outbreaks focus on reducing the risk of the introduction of new pathogens in the rearing facilities, the establishment of cleaning routines, and the maintenance of stable rearing conditions as reviewed by Eilenberg *et al.* (2018). Another method proposed for the mitigation of insect pathogens is oral feeding with probiotics (Savio *et al.*, 2022). Similarly, chemical substances such as formaldehyde have been historically used as a disinfectant and sterilizing agent in insect mass‐rearing facilities, since they help control the growth of bacteria, viruses and fungi (Toth Jr, 2009). The delivery of antiviral compounds has also shown promising results for the mitigation of viral outbreaks (Parker, 2005; Abd‐Alla *et al.*, 2012). For instance, ribavirin lowered the acquisition of Rice stripe tenuivirus in the true bug *Laodelphax striatellus* (Hajano *et al.*, 2020).

In this study, we orally fed medfly larvae with a chemical disinfectant (formaldehyde) and an antiviral drug (ribavirin) to assess their effect on viral RNA levels and medfly fitness. Overall, our results broaden the knowledge of virus eradication or containment methodologies that could be applied to reduce the risk of viral outbreaks in insect rearing facilities.

## Materials and methods

### Insects

Experiments were performed using the Vienna 8A (V8A) medfly strain (Hernández‐Pelegrín *et al.*, 2022). The V8A strain is maintained at the mass‐rearing facility in Caudete de las Fuentes (Valencia, Spain) at 25 ± 1 °C, 65% humidity, and 14/10 h light/dark cycles (Porras *et al.*, 2020; Plá *et al.*, 2021). Three hundred thousand medfly eggs are employed for the development of each subsequent generation and the time required to complete the life cycle is, on average, 30 d. The base larval diet of the V8A strain contains wheat bran, sugar, and brewer's yeast (de Pedro *et al.*, 2021) and adults have water and sugar‐based sources *ad libitum*. Sterile male adults of the V8A strain are released by the state‐owned company Empresa de Transformacion Agraria S.A (Grupo TRAGSA, Valencia, Spain) for the application of SIT. Formaldehyde experiments (November 2020) and ribavirin experiments (May 2021) were performed using the same V8A strain.

### Formaldehyde formulation and delivery through larval diet

Larval diet (density 1 g/mL) was added to 12 × 12 cm Petri dishes (150 g per Petri dish). Ten percent formaldehyde solution (J.T. Baker, Deventer, The Netherlands, 7040) was added to the standard medfly larval diet at two final concentrations: 0.1% (1.5 mL of 10% formaldehyde) and 0.2% (3 mL of 10% formaldehyde), which were selected based on previous literature (Dubey & Das, 2021). For the control treatment, which did not contain formaldehyde, water was added to the standard diet. Two biological replicates were performed per treatment. Eggs from the V8A strain were added to the Petri dishes containing the different larval diets at a final density of 0.67 mL of eggs per kg of diet. Hatching larvae were grown until the pupal stage following the above‐mentioned rearing conditions.

### Ribavirin formulation and delivery through larval diet

Larval diet (density 1 g/mL) was added to 9 × 9 cm Petri dishes (50 g per Petri dish). Ribavirin was acquired in a powder form (Sigma Aldrich, MO, USA, PHR1700), solubilized with distilled water to a final concentration of 10 mg/mL, and stored in darkness at −20 °C. The 10 mg/mL solution of ribavirin was added to 50 g of standard medfly larval diet at two final concentrations: 1 mg of ribavirin per gram of diet (5 mL), and 0.1 mg of ribavirin per gram of diet (500 *µ*L). The two ribavirin concentrations were selected based on previous literature (Parker, 2005; Herrero & Zabalgogeazcoa, 2011; Niu *et al.*, 2016; Espino‐Vázquez *et al.*, 2020). For the control treatment, water was added to the standard diet. Four biological replicates were performed per treatment. Eggs from the V8A strain were added to the Petri dishes containing the different larval diets at a final concentration of 0.67 mL of eggs per kg of diet. Hatching larvae were grown until adulthood following the above‐mentioned rearing conditions.

### Detection and quantification of RNA viruses

Normalized viral levels were obtained for eight pools of 5 pupae per treatment. Pupae were collected (a) on the day with the highest number of pupae to synchronize the analysis of viral levels between treatments, and (b) 2 d after the peak of pupation to assess whether pupa development influenced viral replication. Medfly pupae were separated daily after pupation, kept for 3 d in 3 × 3 cm Petri dishes, and stored at −20 °C before RNA isolation. RNA was isolated from pools of 5 pupae using the TriPure isolation reagent (Roche, Mannheim, Germany, 11667157001) following the manufacturer's instructions. One gram of isolated RNA per sample (pool of 5 pupae) was treated with 1 *µ*L of Invitrogen^TM^ DNAse I (Invitrogen, CA, USA, 18047‐019) to eliminate residues of DNA and reverse transcribed into cDNA using oligo (dT) primers and random hexamers following the Perfect Real Time kit (Takara Bio Inc., Dalian, China, RR037A). The levels of the 13 RNA viruses described for medflies were assessed via RT‐qPCR (StepOnePlus Real‐Time; Applied Biosystems, CA, USA) using virus‐specific primers (Llopis‐Giménez *et al.*, 2017; Hernández‐Pelegrín *et al.*, 2022). Normalized viral RNA levels were calculated by comparing Ct values of RNA viruses and the medfly housekeeping gene *L23a* (accession number: XM_004518966.3), after adjusting for primer efficiency (Herrero *et al.*, 2019).

### Treatment effect on medfly development

The effect of formaldehyde and ribavirin treatment on medfly development was quantified by daily recording the number of pupae (for both types of treatments) and emerged adults (only for ribavirin treatment). Two replicates per treatment were performed for the formaldehyde experiment, and four replicates per treatment for the ribavirin experiment. Per replicate, pupae were counted until the 16th day from egg deposition into the larval diet, to identify potential delays in medfly development. The mean time to pupation was calculated as the average number of days needed to become pupae. Additionally, pupal weight was assessed daily for formaldehyde and ribavirin treatments. The new pupae retrieved each day were counted and weighed using a precision balance. The average pupal weight was calculated per replicate considering the number and weight of the pupae recovered daily. Additionally, 30 pupae per replicate were separated 3 d after pupation for molecular analysis. For the ribavirin treatment, the remaining pupae were maintained until adulthood under the same rearing conditions. Adult emergence was annotated daily until day 23 since eggs deposition, to calculate the percentages of pupae that developed into adults in each treatment. Moreover, the mean time to adult emergence was calculated as the average number of days needed to become adult.

### Visualization and statistical analysis

GraphPad Prism version 8.0.0 124 for Windows (GraphPad Software, San Diego, California, USA, www.graphpad.com) was employed for the visualization of normalized viral levels. Normalized viral RNA levels were transformed to a logarithmic scale for visualization and those RNA viruses with normalized viral levels below 10^−6^ were considered undetectable and marked as “ND” in the figures.

The values obtained during the analysis of medfly fitness (number of pupae, mean time to pupation, pupal weight, adult emergence, and mean time to adult emergence) were submitted to a one‐way ANOVA test to compare the medflies reared on the diverse diet formulations. One‐way ANOVA test was applied with Brown–Forsythe and Welch correction, assuming unequal standard deviations between treatments. Tukey's multiple comparisons test was used for pairwise comparisons between treatments.

For the analysis of normalized viral RNA levels, the normal distribution of the data was checked using a Shapiro–Wilk test, and the homogeneity of variances using a Brown–Forsythe test. Based on the results, a nonparametric Kruskal–Wallis test was applied to determine the differences in normalized viral RNA levels between treatments. Differences between treatments were considered significant when *P* < 0.05.

## Results

### The addition of formaldehyde to the larval diet affects larval development

**Fig. 1:**
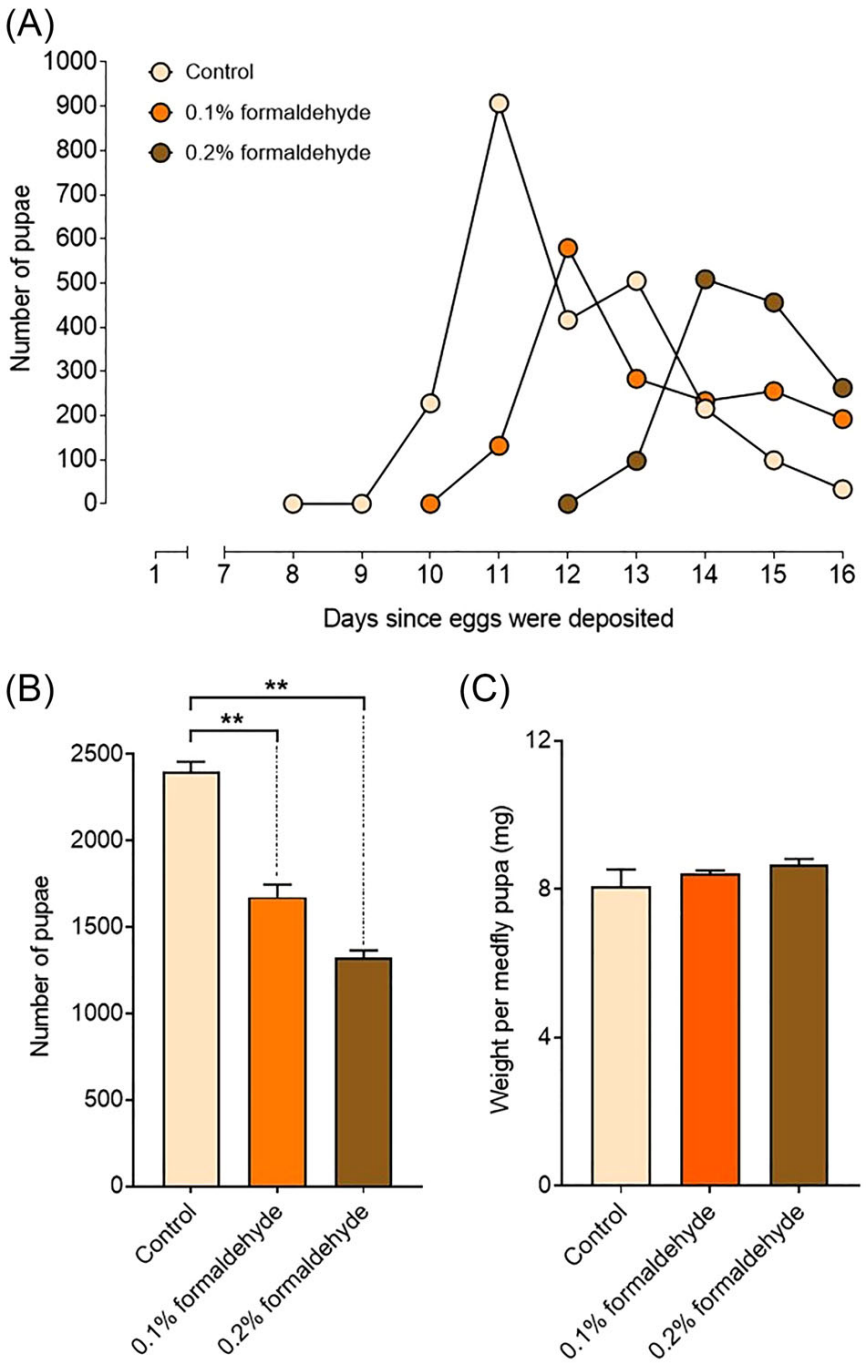
Effects of formaldehyde on medfly development. (A) Dot plot showing the number of pupae collected daily. (B) Bar plot showing the average total number of pupae per treatment (with error bars indicating the standard deviation). Statistical differences in the total number of pupae between the standard diet and the diets supplemented with formaldehyde are displayed in the bar plot (**P < 0.01). (C) Bar plot showing the average weight per pupa per treatment, calculated using the daily number of pupae and pupal weight (with error bars indicating the standard deviation).

The peak of pupation (the day on which the highest number of new pupae were recorded) occurred on day 11 after egg deposition for larvae growing on control diet, on day 12 for the 0.1% formaldehyde diet, and on day 14 for the 0.2% formaldehyde diet (Fig. 1A). This resulted in a shorter mean time to pupation in the standard diet (12.0 ± 0.202 d), than the 0.1% formaldehyde diet (13.3 ± 0.186 d; one‐way ANOVA, *P* = 0.046), and the 0.2% formaldehyde diet (14.7 ± 0.132 d; one‐way ANOVA, *P* = 0.008). Moreover, the delay in the average time to pupation increased at higher formaldehyde concentrations (one‐way ANOVA, *P* = 0.028).

The average number of pupae recovered was 2401 for the control diet, 1673 for the 0.1% formaldehyde diet, and 1324 for the 0.2% formaldehyde diet (Fig. 1B). These results caused significant differences in the number of pupae recovered between the standard diet and the diets containing 0.1% formaldehyde (one‐way ANOVA, *P* = 0.017) or 0.2% formaldehyde (one‐way ANOVA, *P* = 0.004). Instead, no differences were detected between 0.1% and 0.2% formaldehyde treatments (one‐way ANOVA, *P* = 0.581). In terms of pupal weight, no differences were retrieved in the average weight of the pupae between the control diet (8.07 ± 0.457 mg/pupae) and the diets supplemented with 0.1% formaldehyde (8.42 ± 0.080 mg/pupae (one‐way ANOVA, *P* = 0.732) or 0.2% formaldehyde (8.65 ± 0.167 mg/pupae (one‐way ANOVA, *P* = 0.546) (Fig. 1C). Additionally, no difference in pupal weight was detected between 0.1% and 0.2% formaldehyde treatments (one‐way ANOVA, *P* = 0.525),

### Formaldehyde treatment did not affect viral levels

**Fig. 2:**
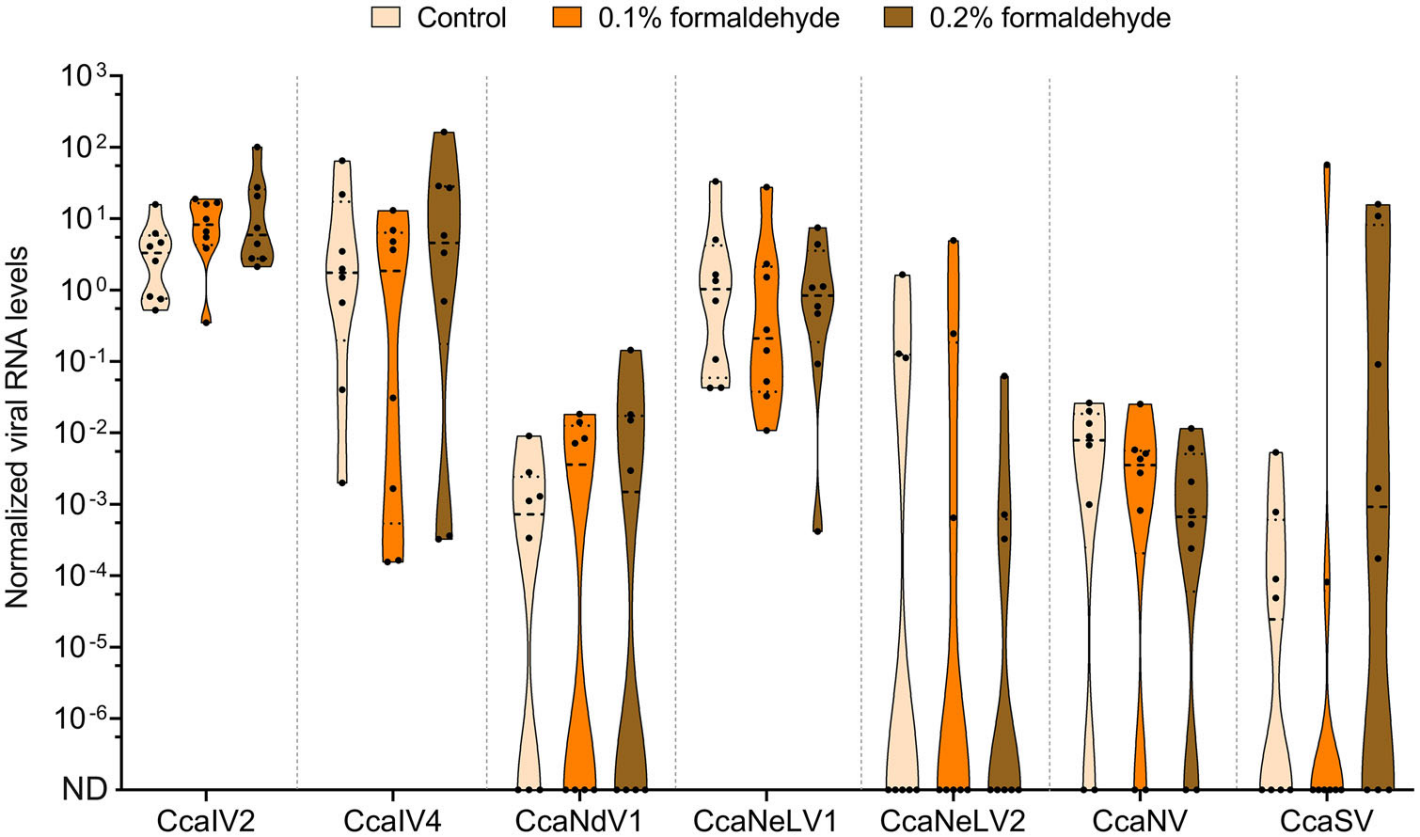
Effect of formaldehyde on RNA virus levels. Violin plot showing the normalized levels of seven RNA viruses in pools of 5 pupae reared on a standard diet or a diet supplemented with 0.1% or 0.2% formaldehyde. Eight replicates are displayed per treatment. ND refers to these samples in which RNA viruses were below detection limits. Normalized RNA levels did not display differences between treatments according to the nonparametric Kruskal–Wallis test.

Out of the 13 known viruses infecting medflies, seven RNA viruses were detected in the V8A strain at the time the experiment with formaldehyde was performed: *Ceratitis capitata* iflavirus 2 (CcaIV2), *Ceratitis capitata* iflavirus 4 (CcaIV4), *Ceratitis capitata* nodavirus 1 (CcaNdV1), *Ceratitis capitata* negev‐like virus 1 (CcaNeLV1), *Ceratitis capitata* negev‐like virus 2 (CcaNeLV2), *Ceratitis capitata* nora virus (CcaNV), and *Ceratitis capitata* sigmavirus (CcaSV) (Fig. 2). Three of them (CcaIV2, CcaIV4, and CcaNeLV1) were detected in all the samples and presented the highest viral RNA levels. Instead, the remaining four viruses (CcaNdV1, CcaNeLV2, CcaNV, and CcaSV) displayed variable levels, and were absent in some of the samples (Fig. 2). No significant differences on the levels of these RNA viruses were identified between medflies reared on the standard diet or the diets supplemented with 0.1% or 0.2% formaldehyde concentrations (Fig. 2). This result evidenced the inefficiency of formaldehyde on reducing the levels of the 7 RNA viruses detected in the V8A strain.

### The addition of ribavirin to the larval diet affects adult emergence

**Fig. 3:**
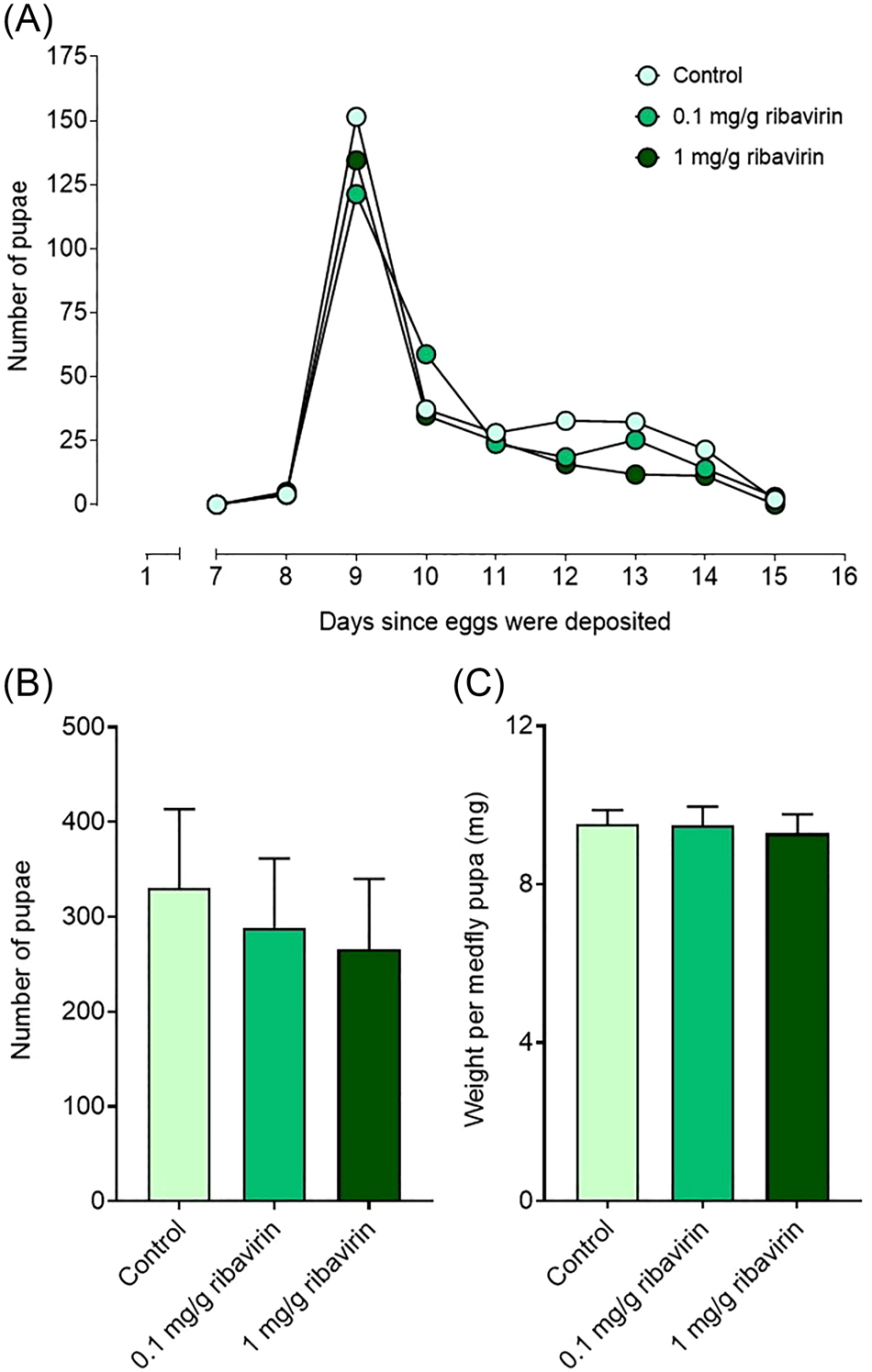
Effects of ribavirin on medfly larva to pupa development. (A) Number of pupae collected daily. (B) Average total number of pupae per treatment (with error bars indicating the standard deviation). (C) Bar plot showing the average weight per pupa per treatment, calculated using the daily number of pupae and pupal weight (with error bars showing the standard deviation).

The peak of pupation occurred on day 9 after egg deposition for the three experimental treatments. This translated in nonsignificant differences in the meantime to pupation between medflies reared on the standard diet (10.5 ± 0.422 d), and the diets supplemented with ribavirin at a 0.1 mg/g concentration (10.4 ± 0.721 d; one‐way ANOVA, *P* = 0.994), or a 1 mg/g concentration (10.3 ± 0.874 d; one‐way ANOVA, *P* = 0.967) (Fig. 3A). Similarly, no differences were detected between the two ribavirin treatments (one‐way ANOVA, *P* = 0.997). On average, the total number of pupae recovered was 331 for the control diet, 289 for the diet with 0.1 mg/g ribavirin, and 266 for the diet with 1 mg/g ribavirin (Fig. 3B). The statistical analysis showed no differences in the number of pupae between the standard diet and the diets supplemented with 0.1 mg/g of ribavirin (one‐way ANOVA, *P* = 0.833) or 1 mg/g of ribavirin (one‐way ANOVA, *P* = 0.602), and between the treatments (one‐way ANOVA, *P* = 0.957) (Fig. 3B). Focusing on pupal weight, no differences were retrieved in the average weight of the pupae reared on the standard diet (9.52 ± 0.361 mg/pupa), and the diets supplemented with ribavirin at 0.1 mg/g (9.48 ± 0.482 mg/pupa; one‐way ANOVA, *P* = 1) or ribavirin at 1 mg/g (9.28 ± 0.487 mg/pupa; one‐way ANOVA, *P* = 0.827) (Fig. 3C). Similarly, no differences were detected between the two ribavirin treatments (one‐way ANOVA, *P* = 0.913).

**Fig. 4:**
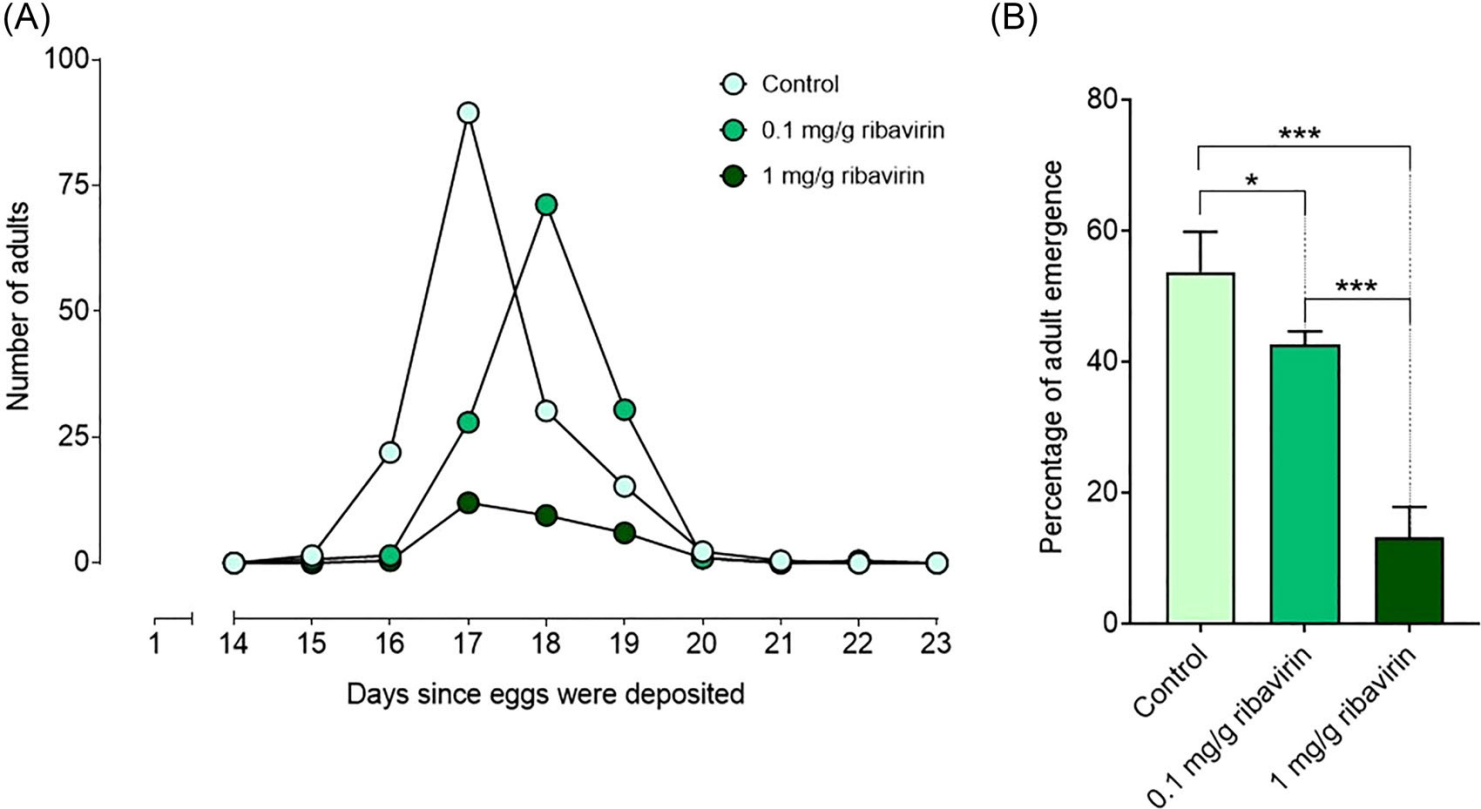
Effects of ribavirin on medfly pupa to adult development. (A) Number of adults collected daily. (B) Total number of emerged adults per treatment. Statistical differences in the percentage of adult emergence between the standard diet and the diets supplemented with ribavirin are displayed in the bar plot (*P < 0.05; ***P < 0.001).

Preliminary experiments revealed a possible effect of the ribavirin on the adult emergence and consequently, we decided to evaluate such parameters for this treatment. To analyze the effect of ribavirin on adult emergence we monitored daily the number of new medfly adults. The mean time to adult emergence did not differ between medflies reared on standard diet (17.3 ± 0.226 d) in comparison to ribavirin 0.1 mg/g diet (17.9 ± 0.356 d; one‐way ANOVA, *P* = 0.114) or ribavirin 1 mg/g diet (18.0 ± 0.718 d; one‐way ANOVA, *P* = 0.349) (Fig. 4A). Differently, the percentage of adult emergence was higher for the standard diet (53.7%) than for the diet with 0.1 mg/g ribavirin (42.6%; one‐way ANOVA, *P* = 0.020), and 1 mg/g ribavirin (13.1%; one‐way ANOVA, *P* < 0.001 (Fig. 4B). Moreover, the negative effect observed on adult emergence was more pronounced at 1 mg/g concentration than 0.1 mg/g concentration (one‐way ANOVA, *P* < 0.001) (Fig. 4B).

### Ribavirin treatment reduced the levels of two RNA viruses

**Fig. 5:**
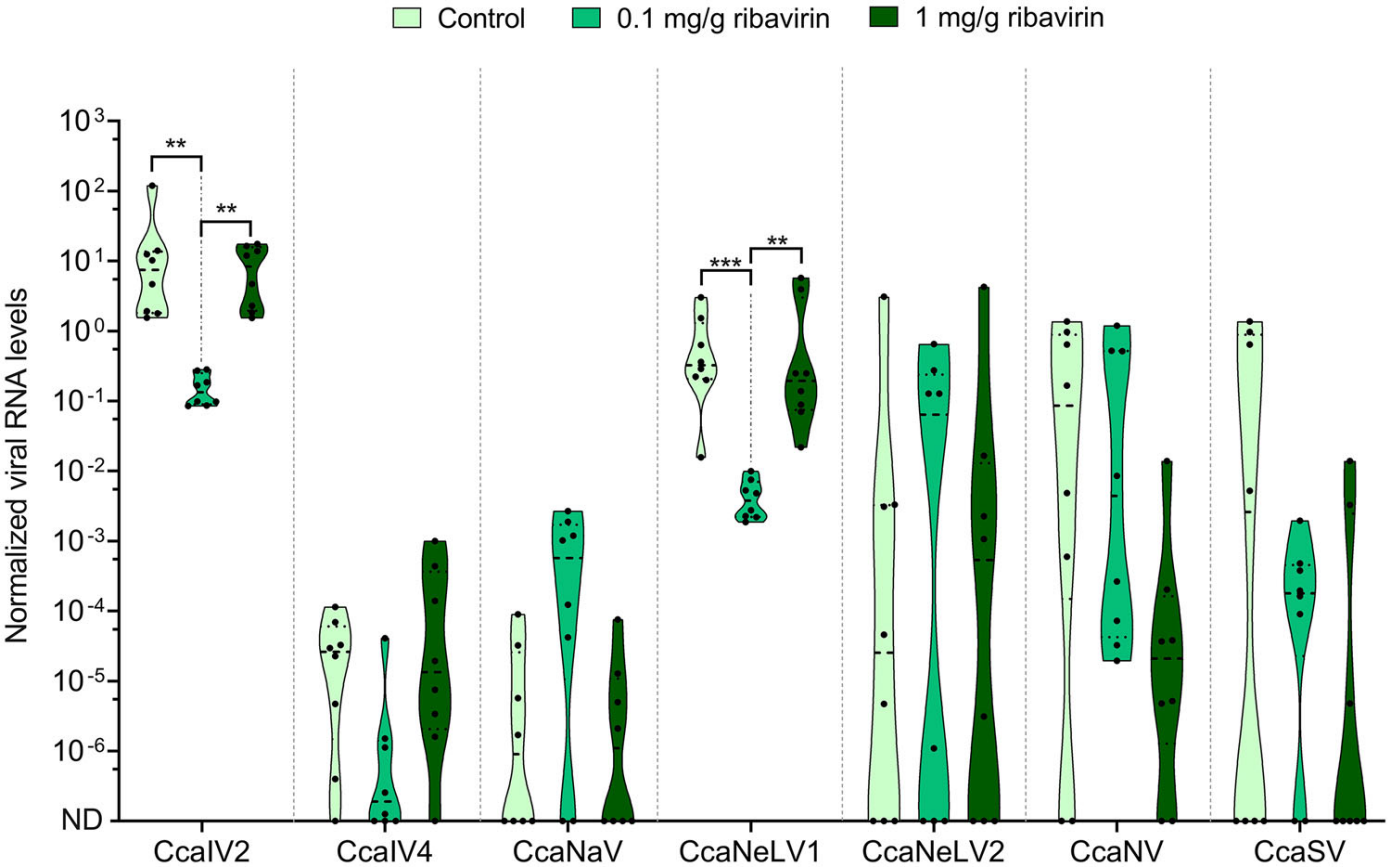
Effect of ribavirin on RNA virus levels. Violin plot showing the normalized levels of seven RNA viruses in pools of 5 pupae reared on standard diet (diet) or diet supplemented with ribavirin at 1 mg/g and 0.1 mg/g concentrations. Eight replicates are displayed per treatment. ND refers to these samples in which RNA viruses were below detection limits. Statistical differences between treatments were calculated using a nonparametric Kruskal–Wallis test and are displayed using asterisks (**P < 0.05; ***P < 0.001).

Seven RNA viruses were detected in the V8A medflies at the time the experiment with ribavirin was performed: CcaIV2, CcaIV4, CcaNeLV1, CcaNeLV2, CcaNV, CcaSV and *Ceratitis capitata* narnavirus 1 (CcaNaV1) (Fig. 5). Two of them (CcaIV2 and CcaNeLV1) were detected in all the samples and presented the highest viral RNA levels. Both viruses displayed higher viral RNA levels in medflies reared on the standard diet and the diet supplemented with ribavirin at a 1 mg/g concentration. However, CcaIV2 and CcaNeLV1 titers were drastically reduced in insects reared on the diet supplemented with ribavirin at a 0.1 mg/g concentration (Fig. 5). In contrast, the addition of ribavirin on medfly larval diet did not affect the levels of the remaining four viruses (CcaIV4, CcaNaV1, CcaNeLV2, CcaNV, and CcaSV), which presented variable levels independently from the diet composition (Fig. 5).

## Discussion

The continuous discovery of insect‐infecting RNA viruses has reshaped our understanding of the viral diversity in insects (Shi *et al.*, 2016; Wu *et al.*, 2020). Most of these viruses remain in a covert state in which no symptoms are observed in the host (Ryabov, 2017). However, in some cases, viral infections cause detrimental effects on host development (Dalmon *et al.*, 2019), which may lead to insect death. Therefore, eradicating certain RNA viruses would benefit the health of the insects, and hence the advent of the insect mass‐rearing industry (Maciel‐Vergara & Ros, 2017). In the medfly, 13 RNA viruses have been discovered (Sharpe *et al.*, 2021; Hernández‐Pelegrín *et al.*, 2022). These viruses are established in different laboratory and field populations distributed worldwide, with variations in the viral repertoire and abundance between populations (Hernández‐Pelegrín *et al.*, 2022). Regarding the function, a single medfly RNA virus, the *Ceratitis capitata* nora virus (CcaNV), has been associated with detrimental effects on host fitness (Hernández‐Pelegrín *et al.*, 2024). However, functional studies exploring the role of other medfly RNA viruses are lacking and one of the main limitations to performing these studies is the absence of natural virus‐free populations.

In this study, two chemical compounds were tested as viral eradication agents following an experimental design that could be reproducible in laboratory and industrial settings, to benefit basic research and insect mass‐rearing. On one hand, we selected formaldehyde based on its widespread use in many insect diets to control microbial growth (Karl *et al.*, 2010) and as a sanitizer in laboratory settings (Lin *et al.*, 2020). Focusing on viral eradication, the addition of formaldehyde inactivated, under specific conditions, the proliferation of two DNA viruses (Vacciniva virus and Human adenovirus) and one RNA virus (Murine norovirus) in mouse and human cells (Möller *et al.*, 2015). Altogether, these results led us to hypothesize that formaldehyde might exhibit antiviral effects in medfly larvae, potentially reducing viral levels (Ricke *et al.*, 2019). However, the addition of formaldehyde into the larval diet did not reduce RNA viral titers while it negatively affected the medfly development. Two plausible explanations for the ineffectiveness of formaldehyde in reducing RNA virus levels could be the delivery method or its concentration. On one side, formaldehyde was added to the larval diet to make this protocol applicable to the mass‐rearing settings (Huynh *et al.*, 2021). On the other hand, formaldehyde concentrations were selected based on previous literature (Dubey & Das, 2021). Further research will be needed to evaluate whether other formaldehyde concentrations and delivery methods may impact viral levels. In any case, the observed adverse effects on pupal development prompt a critical re‐evaluation of the use of formaldehyde in experimental setups involving medflies. Moreover, it is essential to acknowledge the broader implications of formaldehyde use since it is known to be a contaminant and a recognized carcinogenic agent (Dubey & Das, 2021; Protano *et al.*, 2021). Therefore, substituting formaldehyde with alternative and narrow spectra sanitizers that do not pose health risks to both experimental organisms and researchers should be a priority.

Ribavirin was selected based on previous reports demonstrating its potential to reduce viral RNA levels in other insect and fungal systems. For instance, the addition of ribavirin in monosporic cultures of the entomopathogenic fungi *Tolypocladium cyindrosporum* resulted in the eradication of the double strand RNA virus *T. cylindrosporum* virus 1 (Herrero & Zabalgogeazcoa, 2011). In medflies, the addition of 0.1 mg/g of ribavirin into the larval diet significantly decreased the levels of CcaIV2 and CcaNeLV1. These were the only two viruses detected in all the samples under analysis and at higher viral RNA levels. In fact, CcaIV2 and CceNeLV1 have been previously defined as the core RNA virome of the medfly, since they were detected at 100% prevalence in samples collected worldwide (Hernández‐Pelegrín *et al.*, 2022). The widespread presence of CcaIV2 may be explained by its efficient vertical transmission, which occurs both maternally and paternally (Hernández‐Pelegrín *et al.*, 2024), while CcaNelV1 transmission remains unknown. Despite the reduction in CcaIV2 and CcaNelV1 levels observed at 0.1 mg/g ribavirin concentration, this effect was not concentration‐dependent and disappeared at 1 mg/g concentration. The two ribavirin concentrations tested in our experiment were selected based on previous literature. However, testing different concentrations of ribavirin will contribute to understanding its potential for the eradication of RNA viruses without compromising insect fitness.

The absence of dose–response on viral eradication may be explained by the mode of action of ribavirin. Ribavirin is a nucleoside analogue with a broad spectrum of antiviral activity (Parker, 2005). The addition of ribavirin has been related to immunomodulatory effects (Kupke *et al.*, 2023), which may be dysregulated at higher ribavirin doses leading to an impaired immune response against the RNA viruses. Another mode of action proposed for ribavirin is the disruption of the sugar transporter 6 from plants, which is required for viral transmission. Following this mechanism, ribavirin reduced the viral accumulation and transmission of rice stripe tenuivirus (Hajano *et al.*, 2020). In medflies, the addition of ribavirin delayed adult emergence and reduced the number of emerging adults in a dose–response manner. Therefore, the potential adverse effects associated with ribavirin exposure discourage its application in mass‐rearing systems despite the observed reduction in CcaIV2 and CcaNeLV1 levels. Moreover, it remains unclear whether the eradication of these two viruses would positively or negatively affect medfly fitness. For instance, CcaIV2 levels did not vary in a group of flies showing an impaired flying response and mating behaviour (Llopis‐Giménez *et al.*, 2017).

Based on our results, additional alternatives will be needed to generate virus‐free colonies to assess the impact of each of the viruses on insect rearing and also on insect performance once released into the field. One promising alternative for viral eradication is the utilization of RNA interference (RNAi), a powerful molecular mechanism that enables the targeted silencing of specific genes, including those of viruses (Haasnoot *et al.*, 2007). By delivering double‐stranded RNA (dsRNA) corresponding to viral genes into insects, it is possible to trigger RNAi‐mediated degradation of the viral RNA, thereby inhibiting viral replication and spread (Agrawal *et al.*, 2003). This strategy has shown success in silencing various viruses in insects, demonstrating its potential as a viable alternative for virus control in mass‐rearing environments (Niu *et al.*, 2023). Alternatively, random virus segregation can be exploited to generate isofemale lines as previously reported in the Queensland fruit fly, *Bactrocera tryoni* (Morrow *et al.*, 2023).

Overall, our investigation explored the potential of a chemical disinfectant (formaldehyde) and an antiviral drug (ribavirin) for the eradication of RNA viruses. While formaldehyde treatment failed to reduce the viral levels, the addition of ribavirin at 0.1 mg/g concentration effectively reduced the levels of two widespread RNA viruses. Although the reduction in adult emergence caused by the ribavirin treatment questions its applicability in medfly mass‐rearing facilities, our results demonstrated its potential to generate virus‐free medfly strains that would contribute to the study of the virus‐host interactions.

## Disclosure

The authors declare that they have no competing interests.
